# Structural Bridges through Fold Space

**DOI:** 10.1371/journal.pcbi.1004466

**Published:** 2015-09-15

**Authors:** Hannah Edwards, Charlotte M. Deane

**Affiliations:** Department of Statistics, University of Oxford, Oxford, United Kingdom; Wake Forest University, UNITED STATES

## Abstract

Several protein structure classification schemes exist that partition the protein universe into structural units called folds. Yet these schemes do not discuss how these units sit relative to each other in a global structure space. In this paper we construct networks that describe such global relationships between folds in the form of structural bridges. We generate these networks using four different structural alignment methods across multiple score thresholds. The networks constructed using the different methods remain a similar distance apart regardless of the probability threshold defining a structural bridge. This suggests that at least some structural bridges are method specific and that any attempt to build a picture of structural space should not be reliant on a single structural superposition method. Despite these differences all representations agree on an organisation of fold space into five principal community structures: all-*α*, all-*β* sandwiches, all-*β* barrels, *α*/*β* and *α* + *β*. We project estimated fold ages onto the networks and find that not only are the pairings of unconnected folds associated with higher age differences than bridged folds, but this difference increases with the number of networks displaying an edge. We also examine different centrality measures for folds within the networks and how these relate to fold age. While these measures interpret the central core of fold space in varied ways they all identify the disposition of ancestral folds to fall within this core and that of the more recently evolved structures to provide the peripheral landscape. These findings suggest that evolutionary information is encoded along these structural bridges. Finally, we identify four highly central pivotal folds representing dominant topological features which act as key attractors within our landscapes.

## Author Summary

Folds are considered to be the structural units which make up the protein universe. Structural classification schemes focus on the assignment and organisation of protein domains into folds. However, they do not suggest how different folds might relate to one another in a global way. We introduce the concept of bridges through fold space: significant similarities between these units. We consider four alignment methods and a dynamic approach to placing these bridges. A greater consensus between these methods cannot be achieved by simply increasing the stringency with which edges are assigned. Instead, we emphasise the importance of considering consensus maps and only report results where there is agreement across all networks. It is possible that a study of the bridges may reveal evolutionary relationships. Based on a phylogenetic analysis of structures, we find that bridges consistently fall between folds which evolved at similar times. Moreover, the landscapes all consist of a core of older folds, with younger structures more often seen at the periphery. Finally we identify four pivotal folds in the landscapes. They contain topological motifs which unite disparate regions of fold space.

## Introduction

The vast repertoire of proteins which exist in nature are testament to billions of years of evolutionary change. The nature of their relationships and how these have evolved are questions which continue to fascinate the scientific community [1–4]. Protein structure classification schemes such as SCOP [5] and CATH [6] partition the protein universe into different structural units known as folds or topologies. Yet relationships between these folds and topologies, and how they sit relative to each other in a global structure space, are largely undiscussed by these schemes. For example, it is highly unlikely that the current repertoire of folds evolved independently of each other [7]. The evolutionary trajectory of new folds may well be through the adaptation of already existing structures. In fact, recent studies have uncovered such distant relationships between different fold units [8]. This concept has implications for protein structure classification, and more broadly within the field of protein design. A global view of the protein universe which incorporates inter-fold relationships can provide examples of efficient and evolutionary viable transitions between very different structures. More particularly, how this universe has, and continues to, evolve can be used to simulate directed evolution approaches to protein design [9, 10].

Different techniques have previously been explored in order to generate global representations of protein structure space (see, for example, [11]). Commonly, these approaches utilise structural similarities between protein domains, which produce complex, multi-dimensional data structures. The process of deriving a global landscape from these data can vary and will inevitably involve assumptions about the nature of the underlying relationships and the extent to which structural alignments can reproduce them. For example, using multi-dimensional scaling or principal component analysis can produce lower dimensional embeddings of an array of similarity scores [12–21]. In these spaces, two or three dimensional maps can be visualised which approximate the similarity between any two structures as closely as possible by their distance on the reduced axes. An alternative is to use networks to capture relationships resulting from significant alignments [13, 22–30]. Unlike multidimensional scaling approaches, network constructions do not assume that structural similarity between protein domains is transitive [11]. On the other hand, they do require a score threshold to be set: above which an alignment is considered significant.

Networks and embeddings can both be built using a variety of inputs to the similarity score [14, 27, 30]. For example, sequence information can be used to provide the similarity score or supplement structural alignments [12, 14, 15, 29, 31], as can functional annotations [24, 25, 32].

Despite the fact that the above studies construct visualisations of the protein universe using a wide range of different methods, they present a generally consistent picture of this space. In particular, a striking partition between structures based on their secondary structure content is immediately evident [12–14, 17, 21, 26, 29, 32]. Broadly speaking, this partition agrees with the class level classifications of SCOP and CATH, which consist of all-*α* domains, all-*β* domains and mixed *αβ* domains. In SCOP, mixed domains are further split into the parallel stranded *α*/*β* class and the anti-parallel *α* + *β* class. Protein space representations reveal interesting relationships within and between these groups. Several studies comment on the densely clustered group of *α*/*β* structures [26, 31, 32], and the more dissipated *α* + *β* structures [12, 29], with all-*α* and all-*β* domains tending to congregate in between these two extremes [26]. Secondary structure seems to be one of the dominant forces within these spaces. Even when sequence signal alone is used to determine the global landscape, the main secondary structure classes, as well as other classes such as membrane proteins, divide along secondary structure lines [29]. Functional studies also point to the importance of secondary structure. For example, a recent paper exposed a functionally diverse region, at the centre of structure space, which largely overlaps with the *α*/*β* cluster [32].

Visualisations of structure space can also allow us to consider the distribution of protein domains across this space. In particular, highly dense and connected portions of the space can be used as evidence for a continuous landscape [28, 33–35]. This conclusion has implications for the very foundations of our understanding of proteins and their evolution. A continuous protein space indicates that a partitioning of protein structures into folds and topologies is itself meaningless, as these represent discrete units of structure. The initial motivation behind the concept of a discrete fold derives from the fact that structure is a highly conserved property during evolutionary change [36]. As highly correlated to its function, the structure of a protein tends to constrain the variation which is tolerable for that domain to remain operational. The abundant structural similarities between different folds however, have stimulated a debate as to whether this is truly the case [33, 37, 38]. A third view has also developed, that protein space displays both discrete and continuous characteristics [31, 33, 34, 39]. In particular, it has been argued that discrete and continuous paradigms of fold space do not necessarily contradict one another but form complementary descriptions of the evolutionary and structural landscapes respectively [39]. This distinction between structural and evolutionary relationships has also been implemented in the new SCOP2 prototype [40], which separates the hierarchical structure of traditional SCOP where evolutionary units superfamilies are contained within structural units of folds into two distinct categories. It is this dual view of fold space that we will adopt here, and in particular, supplement the discrete classification of domains into folds with summaries of their geometric similarities to other folds to establish a global landscape within which these folds sit. In doing so, it is important to note the assumptions this model makes. The first, which has been stated above, is that there is a duality within the underlying dynamics of the space, where both fold classifications and structural alignments between different folds are meaningful. The second is that the methods we use to capture the discrete units and the continuous relationships are correct. We use the SCOP classification to capture the collapse of the domain universe to discrete folds. While SCOP is well established in the literature, it is by no means the only such scheme. As we have mentioned, CATH describes a complementary scheme [6]. There are also other structural schemes such as FSSP [41], and purely sequence-based classifications, such as Pfam [42]. Similarly, there is no single established method for structural alignment and there are still many unsolved problems in the field [43, 44].

In this paper we present several possible sets of inter-fold relationships, which we term bridges through fold space. Each set of bridges is visualised as a network over 631 SCOP folds. To build these networks we have used four different structural alignment algorithms and with each several different thresholds of similarity. We find that with all methods, the resultant organisation of structure space is at least a partial relic of the alignment algorithm used to generate it. While structural alignment programs have continued to improve in quality over recent years, generating relevant alignments consistently remains an unsolved problem [44]. By examining the areas of consensus between these maps we can more easily identify features with a higher confidence than relying on a single method in isolation.

We show that such consensus spaces are vital to an appreciation of the underlying structural relationships between folds as, even at stringent threshold, a proportion of edges in a network will always be an artefact of the alignment method. Nevertheless, the different networks agree on a well defined partition of fold space into five principal community structures: all-*α*, all-*β* sandwiches, all-*β* barrels, *α*/*β* and *α* + *β*.

We have previously used a phylogenetic analysis of fold usage to estimate an evolutionary age for different folds [45, 46]. Age estimates relate to the emergence of a fold’s structural ancestor and are guided by its prevalence on a diverse set of completely sequenced genomes from across the tree of life. In a previous publication we found that different age estimates demonstrated particular preferences in terms of the properties exhibited by their fold structures [46]. Projecting these age estimates onto the structure space networks could provide the potential to examine the relationship between structure and evolution in a more global way. To explore this hypothesis, we examine properties of the nodes and edges of these networks in the context of their estimated age. In particular, we examine the difference in the age of two folds connected by bridges. We also look at the distribution of fold ages across the networks and how these relate to the centrality of a fold in the network’s architecture. Finally, we examine four highly pivotal ancient folds, each of which exhibit different topological properties which act as structural attractors between disparate regions of the network spaces.

## Methods

### Domain dataset

Domain coordinate files for structures from the four main SCOP classes (all-*α*, all-*β*, *α*/*β* and *α* + *β*) were taken from the ASTRAL database (version 1.75) and filtered to < 40% sequence identity [47]. To ensure these structures were of sufficient quality we removed any file with an assigned aerospaci score of < 0.4, as suggested by Brenner et al. [47]. Due to the requirements of the structural alignment algorithms the dataset was further refined by omitting structures with only backbone *C*_*α*_ coordinates, and those which contained one or more chain breaks. Chain breaks were assigned using the Bio.PDB module in BioPython where successive *C*_*α*_ atoms were further than 4.3Å apart [48]. This resulted in a dataset of 4,098 domains, comprising 793 from the all-*α* class, 948 classified as all-*β*, 1,215 *α*/*β* domains and 1,142 from *α* + *β*. These domains represent a total of 631 folds.

### Pairwise comparisons

Four different methods were used to generate structural alignments between domains in this dataset. These methods have all been previously published and are available as open source programs or code. They are Mammoth (MAMMOTH) [49], jFatcat (FATCAT) [50], TM-align (TM-ALIGN) [51] and Elastic shape analysis (ESA) [52]. These methods were chosen as computationally efficient yet methodologically dissimilar representatives from the wide array of structural alignment approaches. For each of these methods, 8,394,753=(40982) pairwise comparisons were computed. Each method was run using the default parameters. ESA characterised each domain backbone as a curve of N points, where N is the average length of each pair of domains. FATCAT was run in flexible mode and TM-ALIGN used a TM-score normalised by the average length of the domains.

### Alignment scores

TM-ALIGN measures the strength of each alignment through the TM-score, and ESA generates an elastic metric. However, both MAMMOTH and FATCAT alignments produce multiple similarity scores for each alignment. For example, the MAMMOTH program generates a Z-score, E-value, TM score and PSI score. In these cases we chose the score that maximised the area under the ROC curve, when comparing how well each score correctly identified fold siblings under the SCOP classification. As a result of this analysis MAMMOTH alignments were summarised using the Z-score and FATCAT by the p-value (−ln(*p*)).

### Significant alignments as structural bridges

Networks were constructed from the pairwise comparisons by extracting those entries representing strong similarities between different folds. What constituted a strong similarity was determined by examining each score’s distribution and by assessing its ability to discriminate between SCOP folds. We employed a Bayesian analysis to each score, similar to that outlined in [53]. Explicitly, we considered the posterior probability that two domains were representatives of the same fold (*F* = 1) if their similarity *S* was measured above a candidate threshold $\bar{s}$:

$$\begin{array}{c} P\left(F=1|S>\bar{s}\right)=\frac{P\left(S>\bar{s}|F=1\right)P\left(F=1\right)}{P\left(S>\bar{s}|F=1\right)P\left(F=1\right)+P\left(S>\bar{s}|F=0\right)P\left(F=0\right)} \end{array}$$

**Fig 1:**
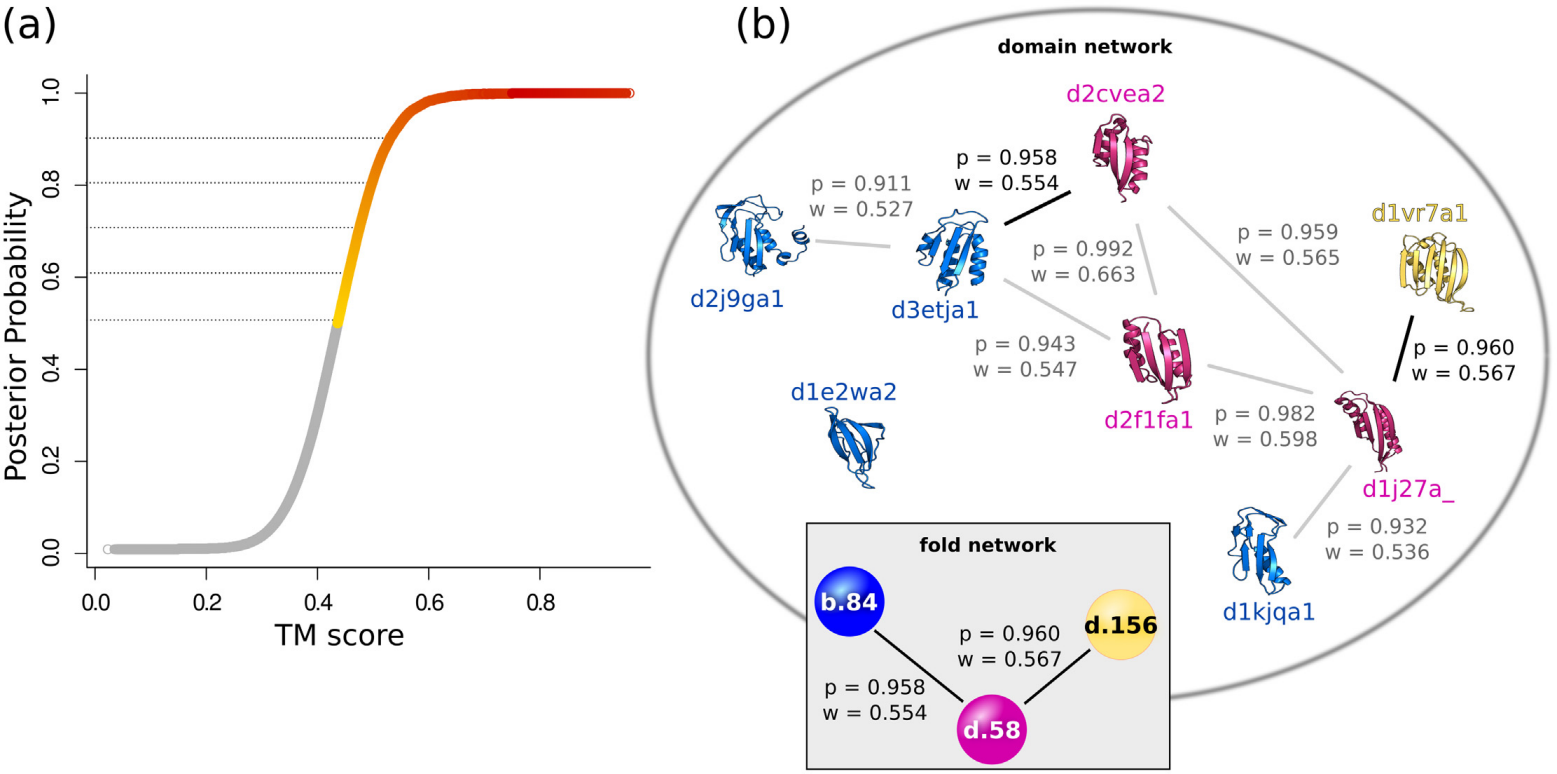
Using the posterior probability to standardise the construction of fold space networks. (a) TM-scores from alignments using TM-ALIGN and their corresponding posterior probabilities. Lines are drawn at probabilities of 0.5, 0.6, 0.7, 0.8 and 0.9. (b) Schematic of the network collapse from the set of domain alignments at a posterior probability threshold of 0.9 to a fold space network. In the final fold network, bridges between folds are defined by the alignment associated with the highest posterior probability. For example, the bridge between fold b.84, shown in blue, and d.58 in pink, derives from the alignment between domains d3etja1 and d2cvea2 with a probability of 0.958 and a TM-score of 0.554.

The prior probabilities *P*(*F* = 1) and *P*(*F* = 0) were assumed to be the proportion of pairs in the domain dataset representing SCOP fold siblings and unrelated domains respectively. The conditional probabilities $P(S>\bar{s}|F)$ were estimated as the proportion of either the set of fold siblings or the set of pairs of unrelated domains in the dataset with similarity scores greater than *s*. Each pairwise alignment was thus associated with a posterior probability between zero and one, based on the relative strength of its score. For example, Fig 1a shows the relationship between the TM-scores of TM-ALIGN alignments and their posterior probabilities. For the purposes of this work we considered only scores associated with a probability ≥ 0.5. We varied this cutoff between 0.5 and 0.9 to show that both the network behaviour and our results remained robust to this choice. As suggested by the FATCAT team, we calculated the significance of comparisons involving all-*α* domains separately to those between the other SCOP classes. This resulted in two different thresholds at each posterior probability: one applicable to alignments involving an all-*α* domain and one for all other alignments. The scores which were used in this analysis and the effective cutoff equivalent to different probabilities are given in Supplementary S1 Table.

### Network construction

Networks of fold relationships were built by collapsing the 4098 × 4098 pairwise array of domains to a 631 × 631 array N of folds. Each entry of this array N(A,B) is characterised by the structural alignment between the pair of representative domains of folds A and B with the highest posterior probability. As the probability threshold decreased from 1 to 0.5 dynamic networks were constructed with folds as nodes and edges between two nodes where any two of their representative domains produced a similarity score which was associated with a probability above the threshold. For probability thresholds of 0.5, 0.6, 0.7, 0.8 and 0.9 static networks were also built. Furthermore at each of these thresholds we constructed consensus networks built from edges between folds appearing in all four networks at that threshold. In total 25 static networks were constructed representing the four alignment methods and their consensus at the five different probability thresholds. Fig 1b shows a simplified schematic of the fold network construction process from pairs of representative domains whose alignments correspond to a posterior probability of at least 0.9.

### Edge weights

Weights were added to each edge and were used to represent a measure of the distance between the folds at its endpoints. The MAMMOTH Z-score and the FATCAT p-value are statistical values and we therefore felt they were inappropriate as quantitative distances between two structures. Instead, we used the TM score as edge weights in both the FATCAT and MAMMOTH networks. TM-scores are generated as part of MAMMOTH’s output, and we calculated an approximate TM-score from FATCAT’s opt_rmsd score and the domain lengths. The TM-score was also used as weights in the TM-ALIGN network and the inverse of the elastic metric was used in the ESA network. Weights in the consensus networks were calculated by first centering weights corresponding to an individual alignment method by dividing them by their mean. Consensus weights were then calculated by averaging the respective normalised weights.

### Network visualisation

Networks were visualised using Cytoscape [54]. Dynamic networks as the posterior probability on edges decreased from 1 to 0.5 were visualised as animations using the DynNetwork plugin. Static visualisations were calculated using a spring embedded layout, while the prefuse layout was used in the dynamic representations.

### Network analysis

Community structures were detected using the Louvain method for non-overlapping communities in weighted networks [55]. Network analysis was performed using the tnet package [56] in R [57]. Shortest path lengths *d*(*i*,*j*) between nodes *i* and *j* were calculated as the minimum sum of reciprocal weights *w*_*hk*_ along the series of edges connecting the two nodes as proposed by Dijkstra [58]:

$$\begin{array}{c} d\left(i,j\right)=\min(\frac{1}{w_{ih}}+\ldots +\frac{1}{w_{hj}}) \end{array}$$

The centrality of a node *i* was calculated using degree (*C*_*D*_(*i*)), closeness (*C*_*C*_(*i*)) and betweenness (*C*_*B*_(*i*)) measures in weighted networks as suggested by Opsahl et al. [56]. Respectively, they are defined:

$$\begin{array}{c} C_{D}\left(i\right)=\sum_{j\in N}w_{ij}\;C_{C}\left(i\right)=\sum_{j\neq i}\frac{1}{d(i,j)}\;C_{B}\left(i\right)=\sum_{j,k\neq i}\frac{\sigma_{jk}\left(i\right)}{\sigma_{jk}} \end{array}$$

where *N* is the set of nodes connected by a single edge to *i*, *w*_*ij*_ is the weight along the edge *ij*, *d*(*i*,*j*) is the shortest path length between nodes *i* and *j* as defined above, *σ*_*jk*_(*i*) is the number of shortest paths between nodes *j* and *k* which go through *i*, and *σ*_*jk*_ is the total number of shortest paths between *j* and *k*. Closeness and Betweenness were calculated for nodes in each connected component separately. Central and peripheral sets of folds were identified for each measure as follows. Central folds were the top 30% of nodes ranked by their centrality measures. Peripheral folds defined by closeness were the bottom 30% of nodes ranked by closeness. Degree and betweenness measures followed a skewed distribution with large numbers of nodes calculated to have very low values and far fewer folds being assigned a high degree or betweenness. Therefore, folds with peripheral degree were those with either one or no neighbour in the network. Similarly, peripheral folds by betweenness were those with a betweenness value of zero.

### Fold age

Evolutionary age estimates were calculated for each fold following the method outlined in [45], and more recently in [46]. These ages use a parsimony algorithm on the predicted fold content of 1014 genomes from across the sequenced tree of life to predict a relative estimate of its structural ancestor. Ages are normalised to lie between zero and one where zero corresponds to a recent ancestor, while an age of one indicates an ancestral fold predicted to exist in the last universal common ancestor. All statistics are calculated assuming an underlying phylogeny of these species as traced from the NCBI taxonomy database [59]. Populations’ age distributions were compared using the Mann Whitney U test [60].

## Results

**Fig 2:**
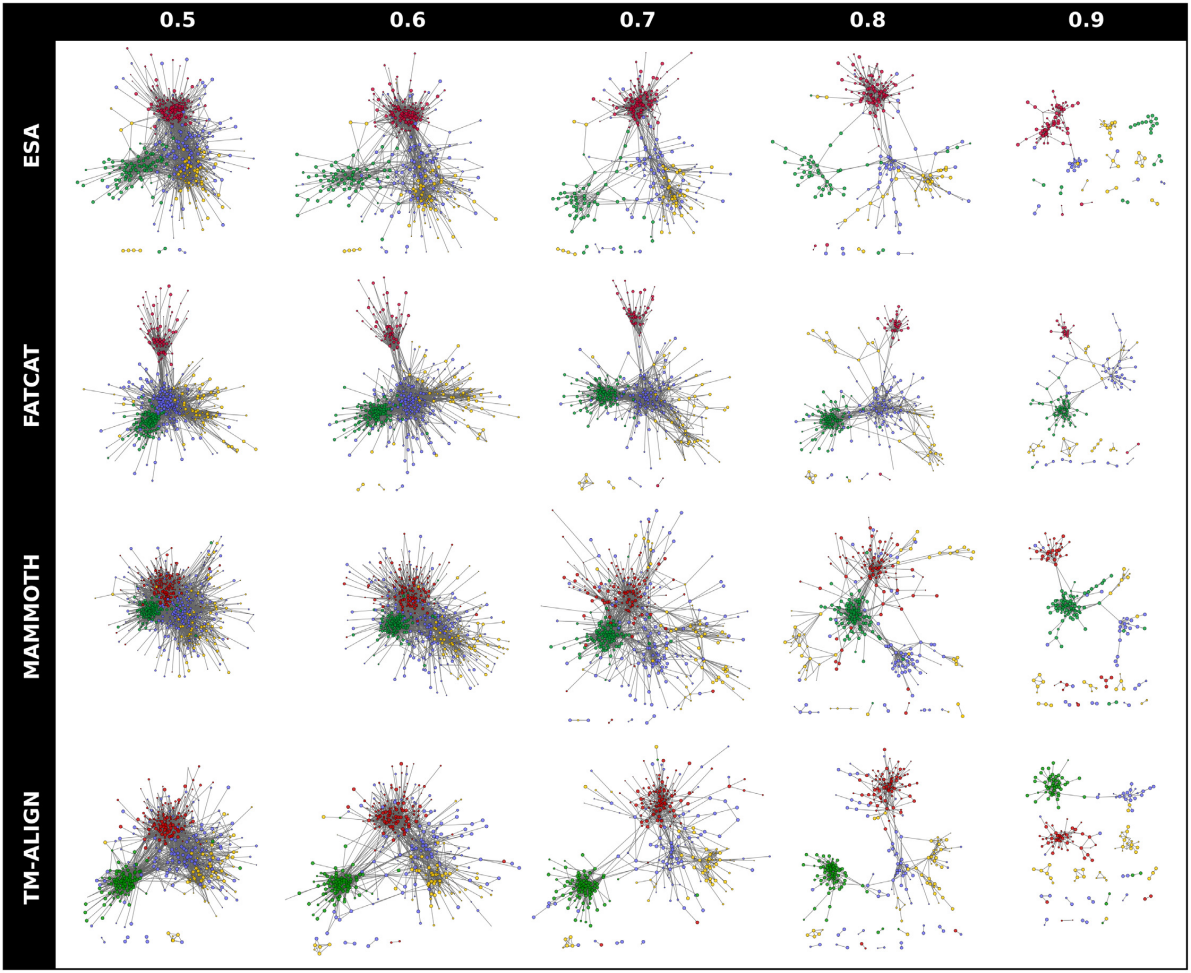
Fold space networks for the four structural alignment methods at increasingly stringent probability cutoffs, ranging from 0.5 to 0.9. Nodes are coloured by their SCOP class (all-*α*: red, all-*β*: yellow, *α*/*β*: green, *α* + *β*: blue) and have a size proportional to their evolutionary age estimate (an age of 0.0 (younger folds) corresponds to a smaller node size, while folds with an age of 1.0 (ancient folds) are larger). Only connected nodes are shown in these representations.

We constructed five dynamic networks representing the structural relationships between 631 well characterised SCOP folds from the four main classes (all-*α*, all-*β*, *α*/*β* and *α* + *β*). Each network summarised the results of the pairwise alignments of 4098 high quality structures representing these folds. Alignments were calculated using four different methods: MAMMOTH, FATCAT, TM-ALIGN and ESA. Four separate networks were built corresponding to each of these methods and one for their consensus, where nodes represented the 631 folds detailed above and edges represented a significant structural similarity resulting from the pairwise alignments. These edges can be seen as structural bridges through fold space, uniquely defining the resultant landscapes. The process of determining significant similarity was standardised across the different methods by the introduction of a posterior probability attached to each method’s score, which quantified its effectiveness in characterising fold level relationships (see Methods for details). Networks were drawn for each method at five different probability thresholds ranging from 0.5 to 0.9, representing increasingly stringent thresholds for determining similarity, and are shown in Fig 2. At each cutoff a consensus network was also built, capturing edges detected by all four methods. In each network bridges were weighted with a similarity measure appropriate to each method.

The networks were further visualised dynamically, as the cutoff for deducing similarity decreased in stringency. Movies displaying dynamic visualisations of these networks as the threshold varies can be found in the Supplementary Information (S1–S5 Videos). They capture both how the landscapes and their consensus are constructed and how robust their organisation is to the probability threshold. These movies can also be found online at http://www.stats.ox.ac.uk/research/proteins/resources#bridges. At high thresholds, the landscapes display an early organisation into disconnected clusters, predominantly of the same class. As the threshold decreases, these components coalesce into a giant component somewhere between a threshold of 0.9 and 0.8. As the threshold decreases still further the overall organisation of the networks remains relatively stable.

### Network collapse

**Fig 3:**
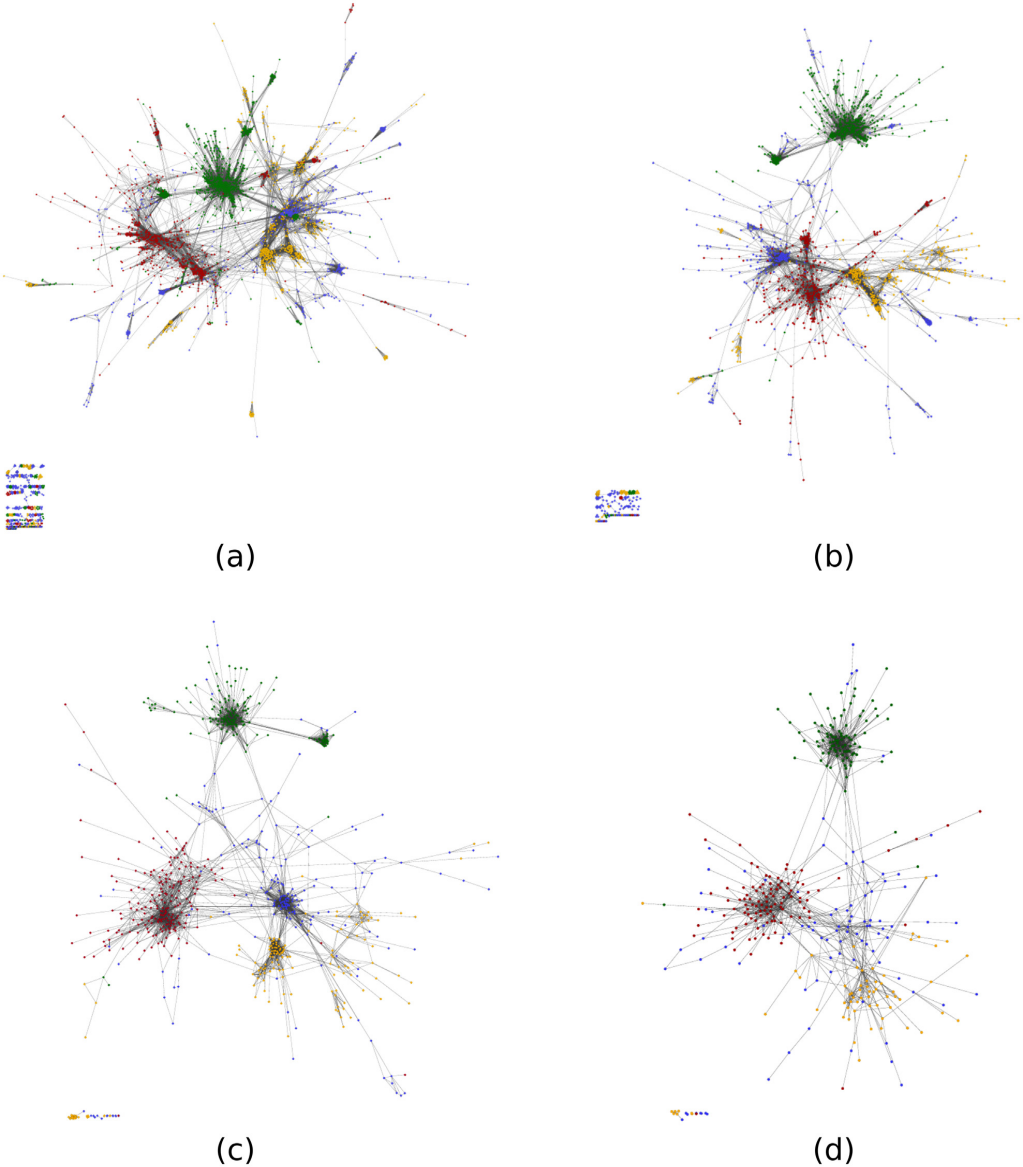
The collapse of the TM-ALIGN network at a probability threshold of 0.7. Networks are drawn for (a) 4098 domains, (b) 1964 families, (c) 1025 superfamilies and (d) 631 folds. For each network, edges are drawn between nodes where any alignment between representative domains yields significant similarity. Classification of domains into families, superfamilies and folds are taken from SCOP. Nodes are coloured by their SCOP class (all-*α*: red, all-*β*: yellow, *α*/*β*: green, *α* + *β*: blue).

The landscapes of structural bridges described above represent similarities at the inter-, rather than the intra-fold level. As such, the method involves a collapse from the set of relationships across 4098 protein domains to those between their 631 different SCOP folds. This process of collapse is an important one as it imposes relationships between domains from an external classification scheme. It is also relevant in the context of comparing our networks to other fold space representations in the literature: some of which consider relationships between domains, and others those between folds. In order to illustrate the stages of the network collapse Fig 3 shows network representations of the TM-ALIGN alignments at a posterior probability threshold of 0.7. These relationships are collapsed first to the SCOP family level, then to superfamilies, and finally to folds. Evident at all stages of collapse is the distinction between the different secondary structure classes. Noticeable too is the relative similarity between the superfamily and fold networks, and a more striking visual difference between the network of domains and that of families. The differences between these early stages of collapse potentially derive from the effects of multiple domains representing a small number of families.

### Structural bridges: Landscape and consensus

For each method the structural bridges at each probability threshold collectively determine a landscape for the global organisation of fold space. Some general network statistics relating to each construction can be found in S1 Fig. As the probability threshold increases, networks become less connected. The number of folds connected to another structure decreases (S1a Fig), and even within connected components, shortest path lengths connecting two folds increase (S1f Fig). The number of edges in the landscapes vary from 5,571 in the MAMMOTH network at a 0.5 threshold to 250 in the ESA network at a threshold of 0.9 (S1b Fig).

An important observation is the significant differences between the alignment algorithms, as well as their areas of agreement. In a single network generated from ESA, MAMMOTH or FATCAT alignments, about 50% of the edges were only identified by that method. For the TM-ALIGN networks, this proportion was somewhat lower at 20–30% of edges (S1c Fig). Moreover, this figure does not improve with increased stringency (i.e. increasing values of the posterior probability). In fact, as the similarity threshold increases, this proportion remains relatively constant, and even increases in the TM-ALIGN and ESA networks. In other words, networks constructed using different alignments remain the same distance apart regardless of similarity threshold. Taken in isolation, a proportion of edges in these networks will always be an artefact of the alignment method, emphasising the importance of considering a consensus network.

### Traversing fold space

As mentioned previously, connected nodes congregate, in most cases, in a single dominant connected component up till a probability threshold > 0.8 (see also S1i Fig). The exception to this is the consensus network, where all-*α* folds are separated from the largest connected component. While there are separate smaller components within the networks (S1h Fig), the vast majority of nodes are either part of a single connected component or are completely unconnected to other structures. This observation supports previous work suggesting that the proportion of unconnected nodes in structure networks sets these structures apart from random models [23], and that fold space can be partitioned into either highly continuous or highly discrete sections [31]. It is also significant regarding the discussion of traversing fold space. A previous study emphasised the short path lengths between structures in fold space as indicative of a continuous space [28]. Within largest connected components we found that average path lengths were less than 5.5 (S1f Fig). While the increase of unconnected nodes is largely responsible for the lack of traversability of networks at higher probability thresholds, it is also interesting to note that, even within connected components, average path lengths between two folds increase. This indicates that the dynamic networks transition from more continuous, connected landscapes at lower probability thresholds to more unconnected spaces at higher thresholds, although both extremes contain densely connected regions and completely unconnected folds.

Previous results have indicated that *α*/*β* structures dominate the highly connected section of fold space [31]. Our results do not find such a dramatic distinction, with all four classes found within the connected component. We do however find that far fewer unconnected folds are *α*/*β* and, within the *α*/*β* cluster shortest path lengths are shorter than those of other classes.

### Secondary structure communities within fold space

**Fig 4:**
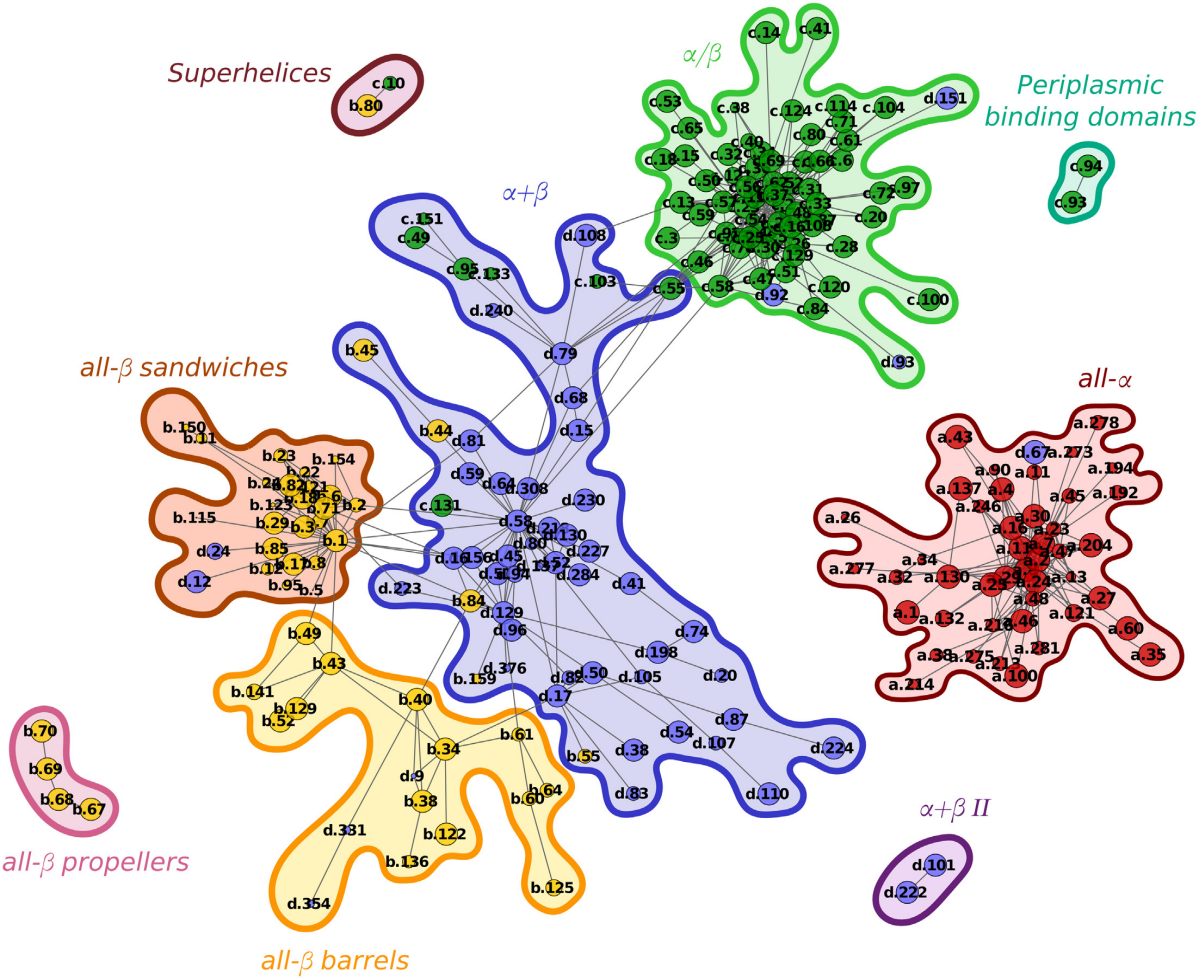
The community structure of the consensus network at a probability threshold of 0.5 as calculated using the Louvain method for weighted networks. Nodes have a size proportional to their age and are coloured by their SCOP class (all-*α*: red, all-*β*: yellow, *α*/*β*: green, *α* + *β*: blue). Clusters are circled and manually assigned labels based on each community’s fold content.

Despite these differences, several properties remain conserved across every landscape. In general, and in concert with previous observations, the networks partition fold space into the four secondary structure classes. The *α*/*β* folds form densely packed clusters, as too, to a lesser degree, do the all-*α* folds. On the other hand, folds with anti-parallel *β* sheets, belonging to the all-*β* and in particular to the *α* + *β* classes are more dissipated throughout the space. Nevertheless, applying a community detection algorithm to these landscapes identifies five predominant communities with a higher density of structural bridges within each group, and sparsely connected externally. These communities can be generally defined as all-*α*, *α*/*β*, *α* + *β*, all-*β* sandwiches and all-*β* barrels by the prevailing population of folds within these clusters. Fig 4 shows the communities in the consensus network which include these five groups along with smaller communities resulting from the smaller connected components of the network. The all-*β* sandwiches and barrels tend to remain partitioned from each other even at the least stringent probability threshold of 0.5 and are often closer in the landscapes to the *α* + *β* community than they are to each other. While the majority of previous visualisations of fold space have noted a four class clustering into SCOP classes [17, 21, 26], one study also saw a division between all-*β* structures [13]. However, in this case *β*-meanders and *β*-zigzags were found to form the basis for this distinction. Meander structures connected to *α* + *β* structures and zigzags included both sandwiches and barrels. This is markedly different from the clusters we find here, where the basis of the division is strictly delineated by a domain’s characterisation as a barrel or sandwich. While some *α* + *β* folds appear within the sandwich and barrel clusters, in these cases they consist of well segregated *α* and *β* regions, with the *β* regions demonstrating the appropriate structural feature.

### Fold ages and the fold networks

Edges in these networks represent, not simply the phenomenon of structural similarity between proteins, but structural bridges between folds: thought to be distinct and separate structural units. We projected fold age estimates, as calculated in [46], onto the folds in each network. These ages estimate the emergence, on a tree of sequenced life, of a fold’s structural ancestor. Each age estimate falls between zero and one, where an age of one represents an ancestral fold emerging at the root of the tree, and an age of zero signifies an ancestor at its leaves. We were thus able to consider the difference in age attributed to each of the bridges in our networks. As edges were undirected we considered the absolute difference in age of the endpoint folds to each edge (bridge). We investigated the distribution of age differences, comparing those of structural bridges to a background distribution of random pairs of dissimilar folds.

**Fig 5:**
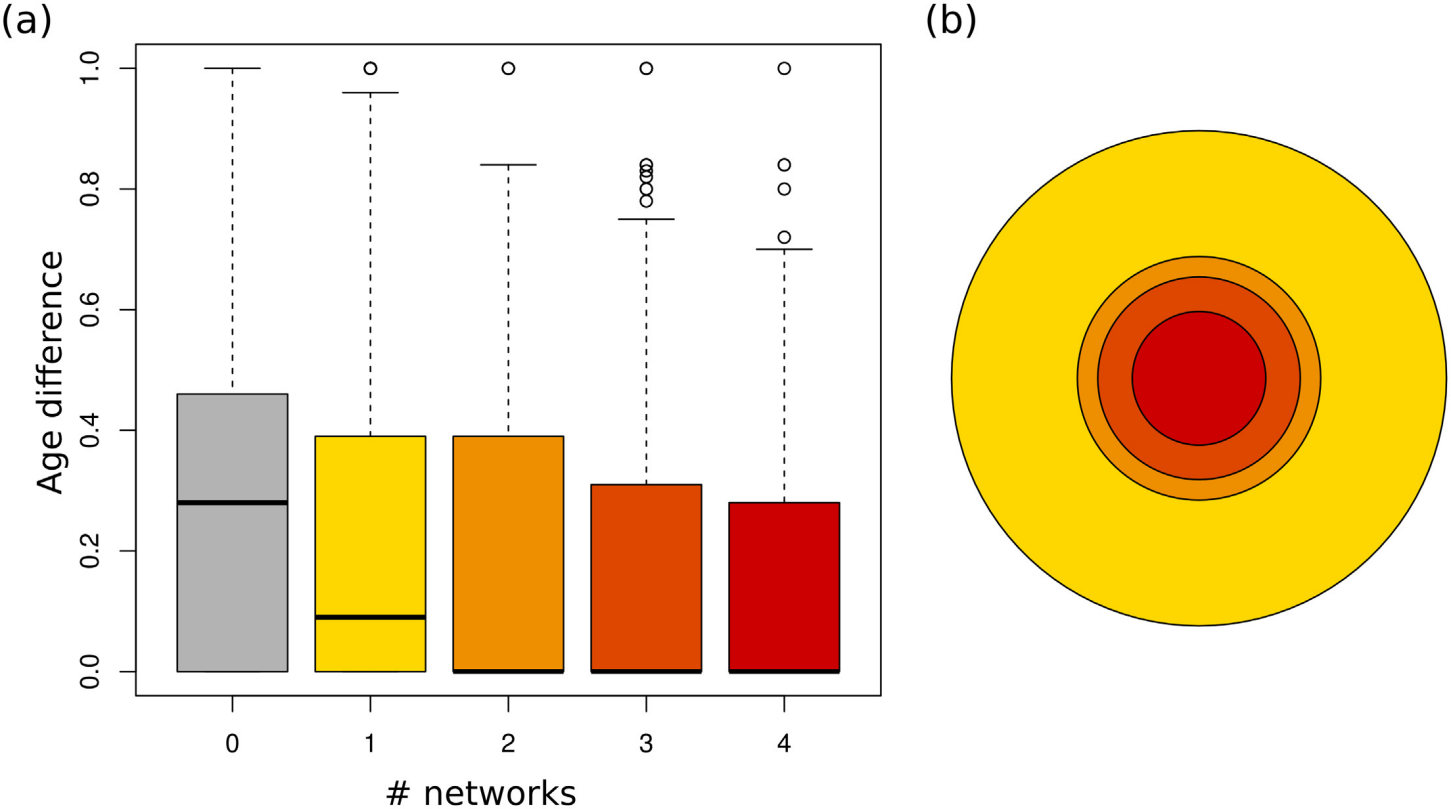
Edges and their age differences in networks with a probability cutoff of 0.6. (a) Boxplot of the age differences between pairs of folds distinguished by the number of alignments methods which assign a bridge to the pair at a probability threshold of 0.6. The median age difference for pairs of unconnected folds is 0.25. This falls to 0 for pairs of folds connected in at least two networks. (b) Concentric circles with an area proportional to the number of edges in each set. There are 9336 edges appearing in just one network, 2250 in just two, 1566 in three, and 678 in all four.

As described above, a large number of these bridges were identified by just a single alignment method so we examined separately the distribution of age differences on edges found on one, two, three and four networks to those found on none. Fig 5a shows a boxplot of these age differences on the set of networks built at a probability cutoff of 0.6. Distributions for the other networks are similar. Not only are the pairings of unconnected folds associated with higher age differences than bridged folds, but this difference increases with the number of networks displaying the edge. Bridges identified by at least two different methods had a median age difference of zero as opposed to the 0.25 of unconnected folds.

### The ancestral core of fold space

**Fig 6:**
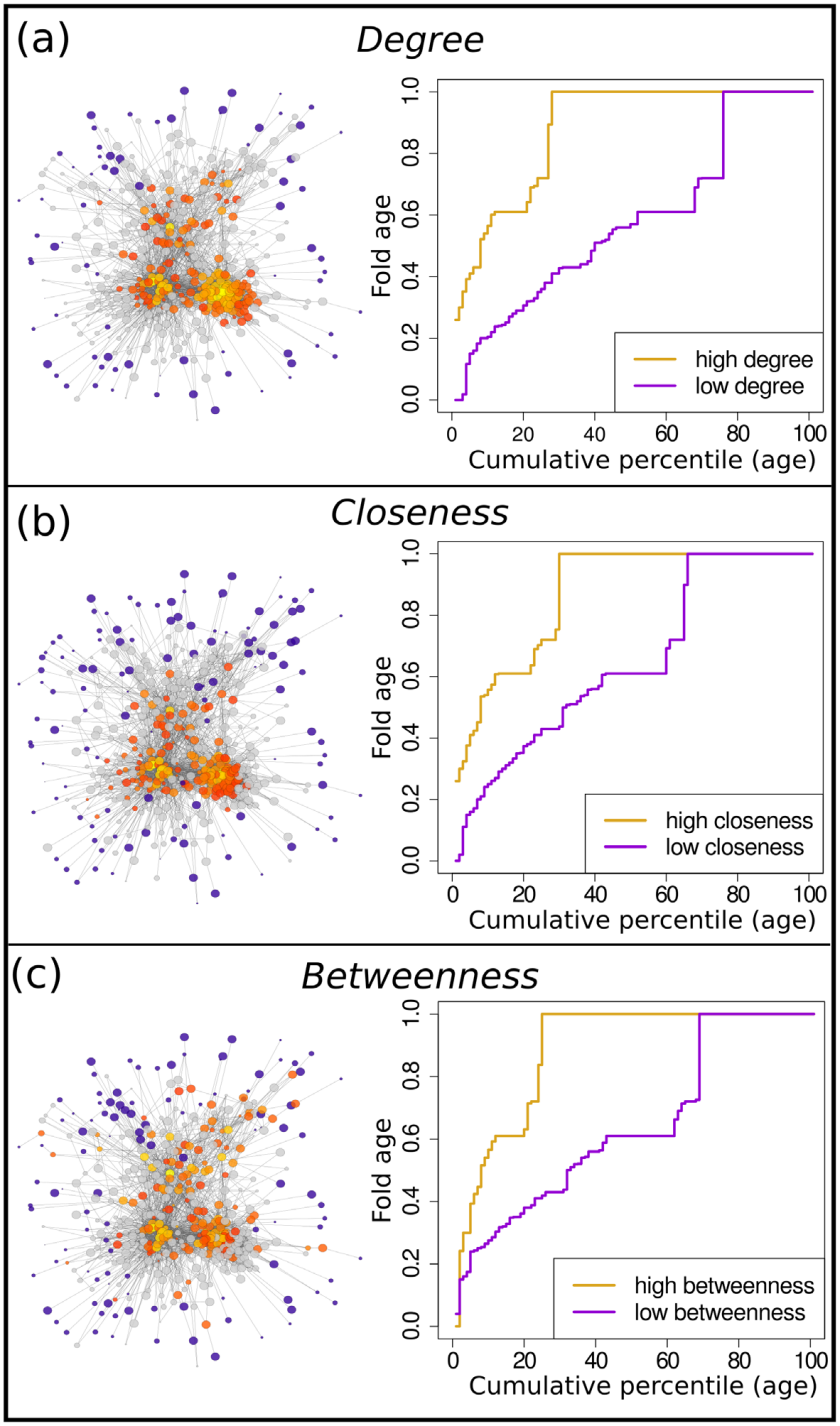
Node centralities in the MAMMOTH network at a probability threshold of 0.6. For each measure of centrality sets of folds with high and low centralities are identified as described in the Methods. These sets are shown projected onto the network. Low centralities are coloured in blue. High centralities are shaded from red (lowest) to yellow (highest). Nodes with intermediate centralities which are not counted as either central or peripheral are shown in grey. Also shown is a cumulative percentile plot for the fold ages of central and peripheral nodes. These graphs show a preference for central nodes to be older than peripheral folds. a) Node centrality by degree. b) Node centrality by closeness. c) Node centrality by betweenness.

The prominence of each fold within these landscapes was calculated using three different centrality measures: degree, closeness and betweenness. For each measure on each network, we identified two populations: central and peripheral folds, and compared the age distributions of these two populations. In every network, including the consensus networks, and by all three of these measures central nodes were found to be significantly older than more peripheral nodes (see S2 Fig). This tendency was true regardless of how we partitioned the nodes. While the three measures produced rankings for the nodes which correlated positively with each other, they all define the concept of centrality slightly differently. Fig 6 illustrates these differences on the MAMMOTH network at a threshold of 0.6.

While these measures interpret the central core of fold space in varied ways they all identify the disposition of ancestral folds to fall within this core and the more recently evolved structures to provide the peripheral landscape.

A previous study found that clusters in structure networks could be associated with functional fingerprints [24]. Based on the assumption that older proteins will be represented by more popular clusters within the domain network, they found that older clusters were matched by a greater heterogeneity in function space. This concept of a functionally diverse ancestral core to structure space was also noted by [32] who found that this region of functional diversity largely derived from a cluster of *α*/*β* domains, which are known to be older folds [19, 45]. We show here that the central core to our networks of structural bridges is significantly older than the peripheral nodes. It is interesting to note that these central, older folds are not in fact dominated by the *α*/*β* class. In fact, folds from the four classes are almost equally represented in these sets. Despite this, our results agree in noting the importance of ancient protein folds within fold space.

### Pivotal folds

The above network centrality analysis further exposed certain pivotal folds, which were calculated as highly central in all networks, including the consensus network. Here we examine four examples of pivotal folds: the long *α*-hairpin (a.2), the Immunoglobulin-like *β*-sandwich fold (b.1), the Flavodoxin-like fold (c.23) and the Ferrodoxin-like fold (d.58). These folds are all ancient, with a fold age of 1.0, and are represented strongly in proteins found right across the tree of life. S3 Fig shows the situation of each pivotal fold within the consensus network at a threshold of 0.5. a.2 and c.23 remain strongly central to the communities of all-*α* and *α*/*β* folds respectively. They are evident as central folds at the highest thresholds of the dynamic networks. On the other hand b.1 and d.58 together connect much more diverse neighbourhoods within the landscapes. In particular, they have an edge between them, and their shared neighbourhood incorporates 61 structural bridges connecting together four distinct communities in the network: the *α*/*β*, all-*β* sandwiches, all-*β* barrels and the *α*/*β* cluster. We visit these folds in more detail in S1 Text. In particular, specific topological features of each fold are specified as instrumental to their highly central positions. Such features include a left-handed *α*-hairpin, the greek key motif and the *α*-*β*-*α* switch.

## Discussion

We have proposed and constructed a dynamic network representation of fold space to capture variations in its organisation resulting from different methodologies and similarity thresholds. While a vast array of different techniques have been applied to visualise the structural organisation of the global protein universe, very little has been done to ensure such landscapes are robust to differences in the alignment methodology which generates them. We have shown that, in terms of network representations using four dissimilar methods, there are several disagreements as to where bridges between different folds in the global space lie. We also found that these disagreements cannot be overcome by simply increasing the threshold at which a structural bridge is determined for each method.

Nevertheless, the four different methods and their consensus networks do converge on certain properties of fold space. In particular, the consistent division between secondary structure classes into five predominant communities: all-*α*, *α*/*β*, *α* + *β*, all-*β* sandwiches and all-*β* barrels. Moreover, folds tend to fall either within a dense and easily traversable connected component, or are completely unconnected. As the probability threshold changes, the balance between these two populations shift as expected, although there remain significant numbers of each at both low and high thresholds.

Structural bridges could exist for a variety of reasons. It is possible they are the result of a misannotation of fold boundaries, or that fold space is wrongly assumed to be discrete. They may also be the result of convergent evolution to a particularly favourable confirmation. They could also represent the structural relic of an evolutionary transition from one fold to another. Whatever their cause, such inter-fold similarities are deserving of further study, to illuminate the overall structure and dynamic of naturally occurring fold space. Moreover, the significant number of these bridges, especially in consensus networks representing the agreement of all four methods, suggests that structural classification, while an important and useful construct, might be a misrepresention of the true nature of the protein universe.

Another feature of the core structure space is the population of ancestral folds at highly central positions within its landscape. Using each different alignment method separately, as well as in consensus, and at different levels of significance, we examined the age distributions of central and peripheral folds. We calculated the centrality of folds as nodes in each network using three different centrality measures, each with a different interpretation of the priority of a node within the landscape. In all these cases, key locations within the landscapes tend to be occupied by older folds than those at the periphery of the space. A previous study identified a functionally diverse core within fold space [61]. This core was predominantly characterised by *α*/*β* folds, which have also been identified as predominantly ancient [19, 45]. The central folds we identify here, on the other hand, represent all four SCOP classes, and form key structural bridges both within and between the class communities. To illustrate this diversity we identified four highly central pivotal folds. These folds represent dominant structural features, such as the greek key motif, the *α*-*β*-*α* switch and the *α* hairpin which act as key attractors within our landscapes.

Structural alignment in general remains an unsolved problem, and much has been written about the inaccuracies of current methodologies. For example a recent study demonstrated a high level of evolutionary inconsistency when comparing several alignment methods, including MAMMOTH, FATCAT and TM-ALIGN [44]. However, despite their limitations, these alignments can give us clues as to a global structure space, in ways in which common classification systems cannot. The representations we have included here cannot be claimed to be accurate depictions of this global space. However, there does appear to be a well defined core to this space where different alignment methods agree on the architecture and general properties of fold space. Moreover, the fact that structural bridges at the heart of this core consensus tend to fall between folds of similar age estimates lends support to the argument that evolutionary information may be encoded along these bridges.

## Supporting Information

S1 Video Dynamic visualisation of the ESA network as the posterior probability decreases from 1.0 to 0.5. Nodes represent folds and edges occur between these folds at the probability value associated with their closest representative domains. Nodes are coloured according to their SCOP class and have a size relative to their estimated fold age.
(MP4)

S2 Video Dynamic visualisation of the FATCAT network as the posterior probability decreases from 1.0 to 0.5. Nodes represent folds and edges occur between these folds at the probability value associated with their closest representative domains. Nodes are coloured according to their SCOP class and have a size relative to their estimated fold age.
(MP4)

S3 Video Dynamic visualisation of the MAMMOTH network as the posterior probability decreases from 1.0 to 0.5. Nodes represent folds and edges occur between these folds at the probability value associated with their closest representative domains. Nodes are coloured according to their SCOP class and have a size relative to their estimated fold age.
(MP4)

S4 Video Dynamic visualisation of the TM-ALIGN network as the posterior probability decreases from 1.0 to 0.5. Nodes represent folds and edges occur between these folds at the probability value associated with their closest representative domains. Nodes are coloured according to their SCOP class and have a size relative to their estimated fold age.
(MP4)

S5 Video Dynamic visualisation of the consensus network as the posterior probability decreases from 1.0 to 0.5. Nodes represent folds and edges occur between these folds at the probability value associated with their closest representative domains. Nodes are coloured according to their SCOP class and have a size relative to their estimated fold age.
(MP4)

S1 Text Pivotal folds within the fold space networks. These four folds were identified as highly central, in terms of degree, closeness and betweenness measures in all the networks of our study, including the consensus networks. Their topologies as well as their neighbours in the consensus network at threshold 0.5 (shown in S3 Fig) are discussed.
(PDF)

S1 Fig Some basic statistics for the four networks as the probability threshold changes. Plots of network statistics for different methods at different thresholds. The statistics are calculated using the tnet package in R. (a) The number of nodes in a network refers to the number of folds connected by at least one bridge. (b) The number of edges is the number of bridges determined as significant by each method at the threshold. (c) Unique edges are edges found in a network at a particular probability threshold which are not found in any other network at that threshold. (d) The density is the proportion of all possible pairs between the set of connected nodes (nodes in (a)) which are bridges. (e) Average neighbours is the average number of bridges connecting a fold in the network. (f) The shortest path between any two nodes is calculated as the smallest sum of weights along bridges forming a path between those folds. The average shortest path for a network is the average of these path lengths across all pairs of nodes in its largest connected component (lcc). (g) The clustering coefficient is the proportion of triplets of connected folds which have bridges connecting all three. (h) Connected components are groups of nodes (> 1 node) which are linked to each other by edges, but which have no edges to nodes outside the component. The number of such components in each network is given here. (i) The number of connected components with at least 20 nodes. This gives an estimate of how many larger components each network consists of.
(TIF)

S2 Fig Plots showing the mean fold age of the central and peripheral folds within each network. These populations were identified for each network as in the Methods. In each case the central nodes are found to be older than the peripheral nodes (significant at the 0.01 level with the Mann-Whitney U test). For simplicity, only points corresponding to networks with one giant component containing all SCOP classes, are included. This is due to the need to consider connected components separately for closeness and betweenness centralities. However, the same signal is seen for the different components of the remaining networks: separate networks at a threshold of 0.9 and consensus networks at all thresholds.
(TIF)

S3 Fig Pivotal nodes and their neighbours in the consensus network with a probability threshold of 0.5. The background consensus network is shown in grey with nodes given a size proportional to their fold age. Pivotal nodes are highlighted as diamonds and coloured according to their SCOP class (a.2: red, b.1: yellow, c.23: green, d.58: blue). Neighbouring folds are coloured according to the pivotal fold they share a bridge with. Folds in orange share a bridge with both b.1 and d.58 and those in purple are connected to c.23 and d.58. Additionally, cartoon representations of representative domains from each pivotal fold demonstrate their topologies.
(TIF)

S1 TableEffective cutoffs for alignment scores at different probability thresholds.For posterior probabilities $P(F=1|S>\bar{s})$ ranging from 0.5 to 0.9, alignment score values $\bar{s}$ were identified as thresholds for network construction. For example, an alignment with a TM-score of 0.5 will translate to a bridge in the TM-ALIGN networks at probability values 0.5 − 0.8 but not in the network corresponding to a probability of 0.9. The elastic metric of ESA’s algorithm is a distance rather than a similarity measure. The cutoffs are therefore upper limits, whereas for the other methods they are lower limits.(TIF)
