# Supplementary material for: Structural Bridges through Fold Space
Source: PLoS Comput Biol. 2015 Sep 15;11(9):e1004466. doi: 10.1371/journal.pcbi.1004466 (PMC4570669; doi:10.1371/journal.pcbi.1004466)
Supplement: S1 Text — These four folds were identified as highly central, in terms of degree, closeness and betweenness measures in all the networks of our study, including the consensus networks. Their topologies as well as their neighbours in the consensus network at threshold 0.5 (shown in S3 Fig) are discussed. (PDF) [file pcbi.1004466.s006.pdf]

Text S1

Pivotal folds within the fold space networks. These four folds were identified as highly central, in terms of degree, closeness and betweenness measures in all the networks of our study, including the consensus networks. Their topologies as well as their neighbours in the consensus network at threshold 0.5 (shown in Fig S3) are discussed further below.

a.2: The long α-hairpin fold consists of two long anti-parallel α-helices connected by a short loop segment with a left-handed twist. In the consensus network shown in Fig S3 a.2 is connected to the majority of folds in the all-α densely clustered group. It is not connected to any fold from another class. In fact, nor is any other all-α fold: the all-α community is a separate connected component in the consensus network. Topologically this fold is relatively simple, however the majority of the alignments resulting in its neighbourhood match both the helical hairpin and also the relative orientations of the two helices brought about by the left-handed twist in a.2.

b.1: The Immunoglobulin-like β-sandwich contains 28 different superfamilies, all similar topologically to the anti-parallel β-sandwich which comprises the core of the antibody, or Immunoglobulin (Ig), domain. This fold consists of two β-sheets with at least one greek key motif connecting strands across the two sheets. In the network in Fig S3 b.1 is connected to a large number of other all-β folds. In the majority of these cases neighbouring folds are β-sandwiches containing a greek key motif. However, b.1 also aligns with two greek key barrel folds at a threshold of 0.5: b.43 (Reductase/isomerase/elongation factor common domain) and b.49 (Domain of alpha and beta subunits of F1 ATP synthase-like). It is these connections which drive the relationship between the communities of all-β sandwiches and barrels in the network. The Ig-like fold also neighbours several jelly roll topologies: b.18 (Galactose-binding domain-like), b.22 (TNF-like), b.85 (Beta-clip) and b.123 (Hypothetical protein TM1070). Finally, b.1 is also connected to seven $\alpha+\beta$ folds across different regions in the network d.12 (Ribosomal proteins S24e, L23 and L15e), d.16 (FAD-linked reductases, C-terminal domain), d.24 (Pili subunits), d.58 (Ferredoxin-like), d.81 (FwdE/GAPDH domain-like), d.129 (TBP-like) and d.156 (S-adenosylmethionine decarboxylase).

c.23: The Flavodoxin-like fold is made up of three layers: a 5-stranded parallel β-sheet packed against a helical layer on both sides. The strand order in the sheet is 21345. In terms of secondary structure, the chain alternates between β-strand units and α-helical units. Thus the helices all run anti-parallel to the strand direction, and parallel to each other. The two helices closest to the N terminus and C terminus respectively form one of the helical layers. The other is formed of the three interior helices. In the consensus network c.23 is connected to the majority of the other α/β folds which make up the densely packed group of this SCOP class. The majority of its neighbouring folds also consist of three layers, with a parallel β-sheet, with a similar strand order, as the second layer. Interestingly a large number of the network siblings of c.23 contain a similar strand ordering of the β-sheet, with strands running consecutively from the centre of the sheet to its edges, first at the N terminal end, then at the C (i.e. of the form 321456). The β-α-β motifs which connect consecutive strands remain largely conserved across these structural siblings, with more variation in the longer linking regions between the non-hydrogen bonded strands. c.23 is also connected to two $\alpha + \beta$ folds: d.79 (Bacillus chorismate mutase-like) and d.92 (Zincin-like). Both of these folds are formed of a mixed β-sheet layer and a helical layer, and contain β-α-β motifs which align well to domains in c.23. They are also both connected to other α/β folds.

d.58: The Ferrodoxin-like fold is one of the most prosperous SCOP folds, containing 40 superfamilies and over 1000 PBD entries. Structurally, it consists of a sandwich: an anti-parallel β-sheet packed against two α-helices. At its core, it contains two repeats of an β-α-β switch motif, where two non-consecutive strands in the sheet are connected via an α-helix running anti-parallel to the strand direction. These two motifs are interlocked together in the fold, involving alternate strands in the β-sheet, and one running from left to right and the other from right to left. In the consensus network shown in Fig S3 d.58 is connected to several other $\alpha + \beta$ folds which also contain two layers and are made up of α-β-α switches. It is also significantly aligned to two α/β folds: c.58 (Aminoacid dehydrogenase-like, N-terminal domain) and c.131 (Peptidyl-tRNA hydrolase II), both of which contain three layers, but also contain β-α-β switches at their core. Moreover, d.58 is further connected to four all-β sandwich domains, including the pivotal fold b.1. In these cases, the two α-helices in the helical layer of b.58 are aligned to the second β-sheet in the sandwich domains.
