# Supplementary figures and images for: Structural Bridges through Fold Space
Source: PLoS Comput Biol. 2015 Sep 15;11(9):e1004466. doi: 10.1371/journal.pcbi.1004466 (PMC4570669; doi:10.1371/journal.pcbi.1004466)

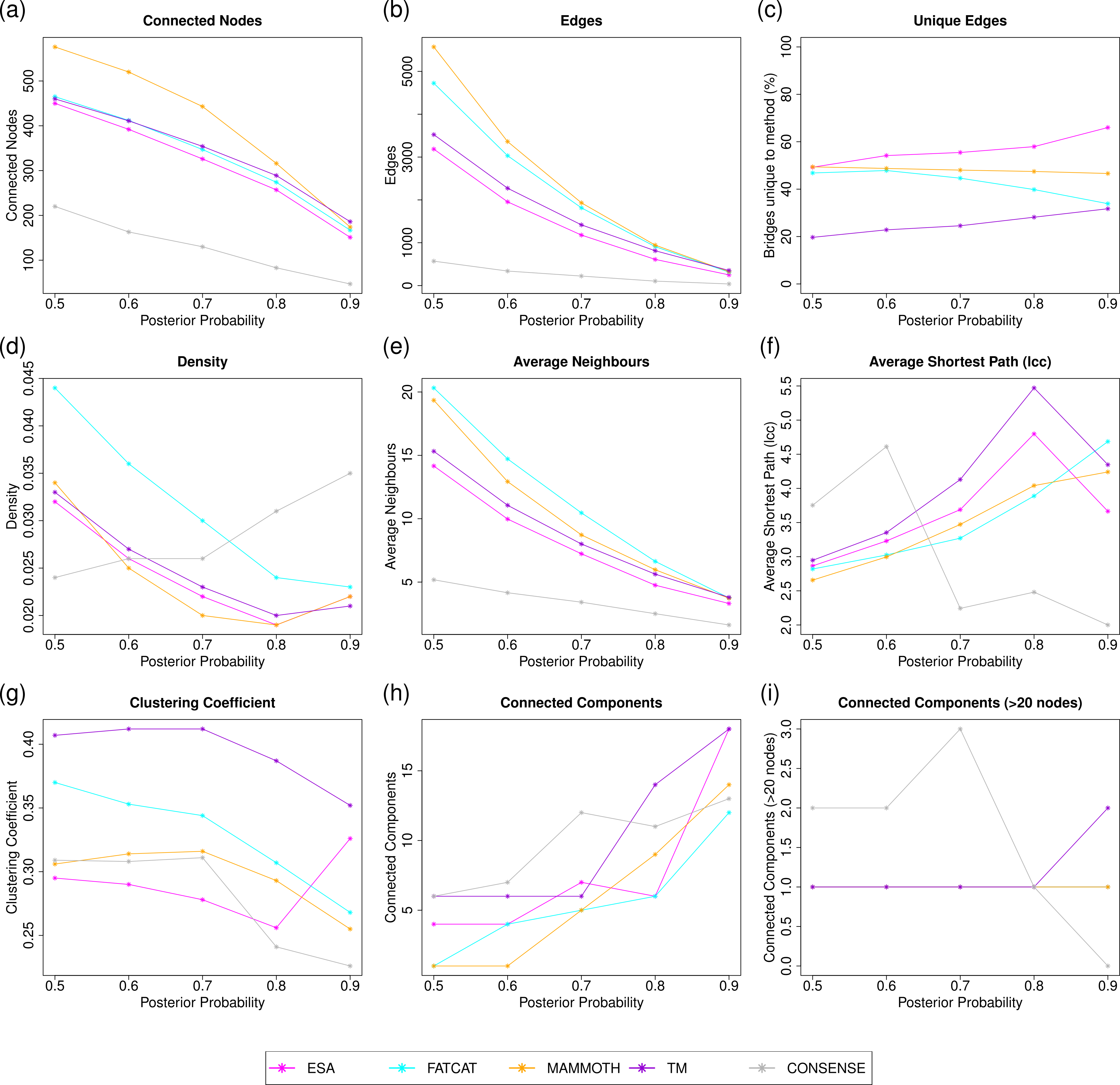

Supplement: S1 Fig — Plots of network statistics for different methods at different thresholds. The statistics are calculated using the tnet package in R. (a) The number of nodes in a network refers to the number of folds connected by at least one bridge. (b) The number of edges is the number of bridges determined as significant by each method at the threshold. (c) Unique edges are edges found in a network at a particular probability threshold which are not found in any other network at that threshold. (d) The density is the proportion of all possible pairs between the set of connected nodes (nodes in (a)) which are bridges. (e) Average neighbours is the average number of bridges connecting a fold in the network. (f) The shortest path between any two nodes is calculated as the smallest sum of weights along bridges forming a path between those folds. The average shortest path for a network is the average of these path lengths across all pairs of nodes in its largest connected component (lcc). (g) The clustering coefficient is the proportion of triplets of connected folds which have bridges connecting all three. (h) Connected components are groups of nodes (> 1 node) which are linked to each other by edges, but which have no edges to nodes outside the component. The number of such components in each network is given here. (i) The number of connected components with at least 20 nodes. This gives an estimate of how many larger components each network consists of. (TIF) [file pcbi.1004466.s007.tif]

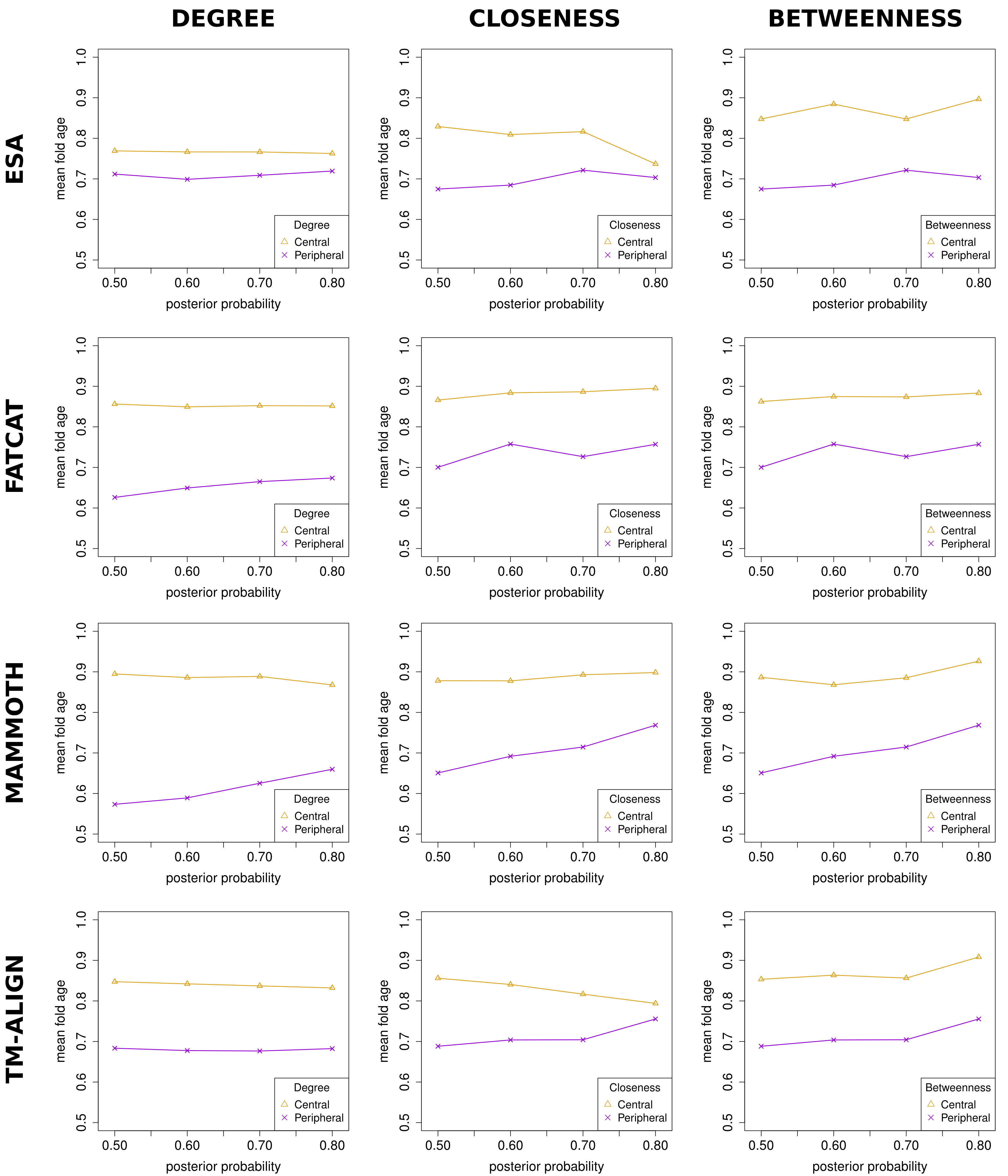

Supplement: S2 Fig — These populations were identified for each network as in the Methods. In each case the central nodes are found to be older than the peripheral nodes (significant at the 0.01 level with the Mann-Whitney U test). For simplicity, only points corresponding to networks with one giant component containing all SCOP classes, are included. This is due to the need to consider connected components separately for closeness and betweenness centralities. However, the same signal is seen for the different components of the remaining networks: separate networks at a threshold of 0.9 and consensus networks at all thresholds. (TIF) [file pcbi.1004466.s008.tif]

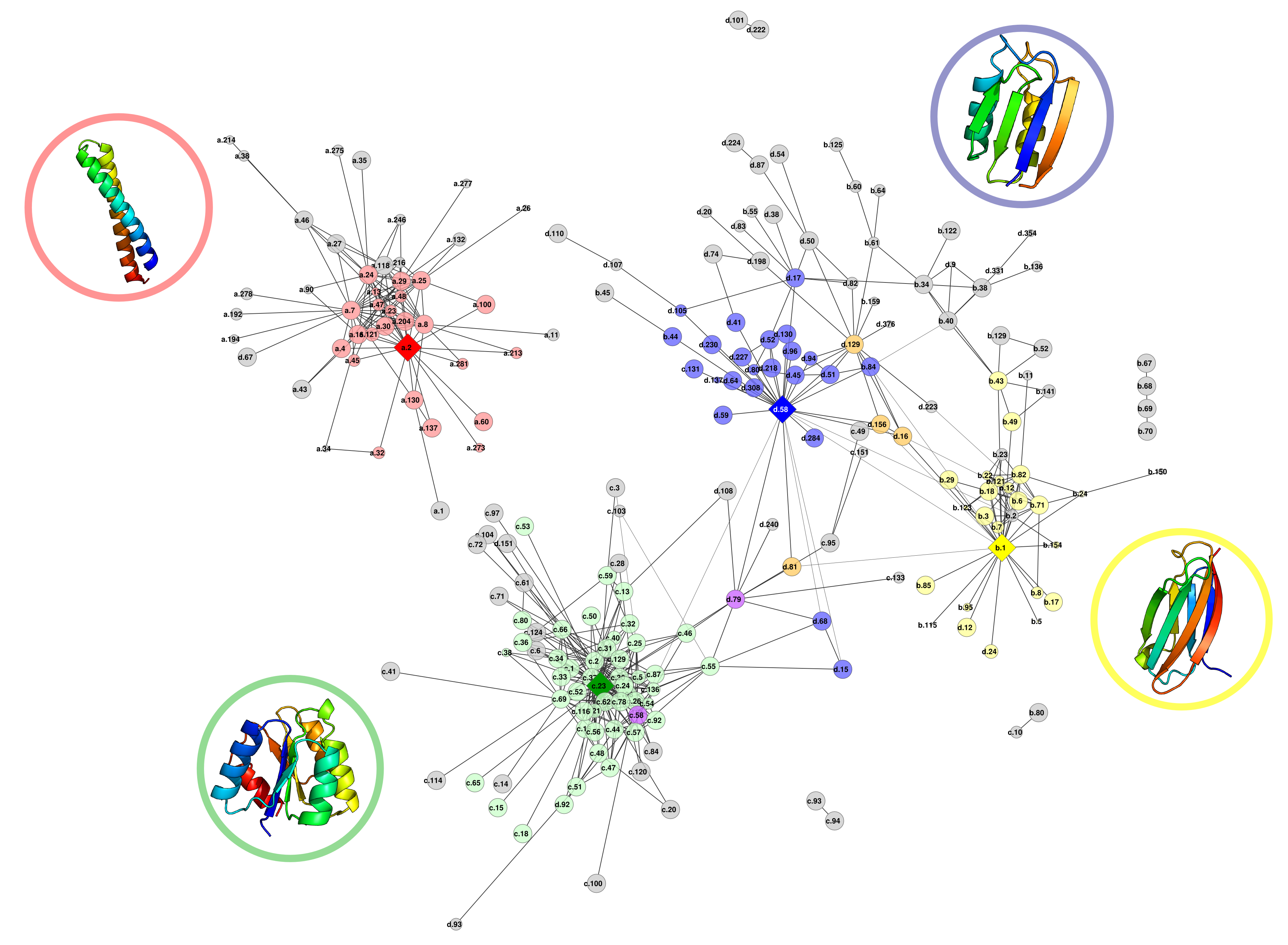

Supplement: S3 Fig — The background consensus network is shown in grey with nodes given a size proportional to their fold age. Pivotal nodes are highlighted as diamonds and coloured according to their SCOP class (a.2: red, b.1: yellow, c.23: green, d.58: blue). Neighbouring folds are coloured according to the pivotal fold they share a bridge with. Folds in orange share a bridge with both b.1 and d.58 and those in purple are connected to c.23 and d.58. Additionally, cartoon representations of representative domains from each pivotal fold demonstrate their topologies. (TIF) [file pcbi.1004466.s009.tif]

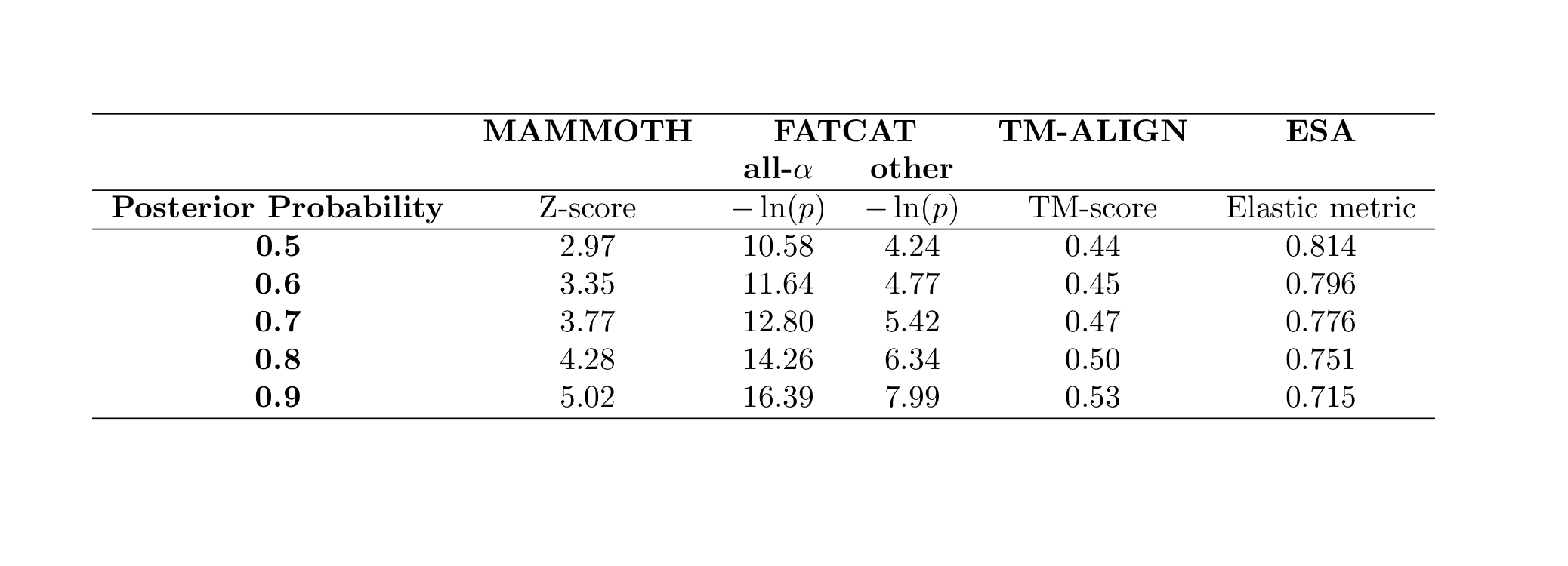

Supplement: S1 Table — For posterior probabilities P(F=1|S>s¯) ranging from 0.5 to 0.9, alignment score values s¯ were identified as thresholds for network construction. For example, an alignment with a TM-score of 0.5 will translate to a bridge in the TM-ALIGN networks at probability values 0.5 − 0.8 but not in the network corresponding to a probability of 0.9. The elastic metric of ESA’s algorithm is a distance rather than a similarity measure. The cutoffs are therefore upper limits, whereas for the other methods they are lower limits. (TIF) [file pcbi.1004466.s010.tif]
